# A supplementary circuit rule-set for the neuronal wiring

**DOI:** 10.3389/fnhum.2013.00170

**Published:** 2013-05-01

**Authors:** Kunjumon I. Vadakkan

**Affiliations:** Division of Neurology, Department of Internal Medicine, Faculty of Medicine, University of ManitobaWinnipeg, MB, Canada

**Keywords:** circuit rules, motor learning, internal sensation, connectome, membrane hemi-fusion, long-term potentiation (LTP), wiring rules

## Abstract

Limitations of known anatomical circuit rules necessitate the identification of supplementary rules. This is essential for explaining how associative sensory stimuli induce nervous system changes that generate internal sensations of memory, concurrent with triggering specific motor activities in response to specific cue stimuli. A candidate mechanism is rapidly reversible, yet stabilizable membrane hemi-fusion formed between the closely apposed postsynaptic membranes of different neurons at locations of convergence of sensory inputs during associative learning. The lateral entry of activity from the cue stimulus-activated postsynapse re-activates the opposite postsynapse through the hemi-fused area and induces the basic units of internal sensation (namely, semblions) as a systems property. Working, short-term and long-term memories can be viewed as functions of the number of re-activatible hemi-fusions present at the time of memory retrieval. Blocking membrane hemi-fusion either by the insertion of the herpes simplex virus (HSV) glycoproteins or by the deposition of insoluble intermediates of amyloid protein in the inter-postsynaptic extracellular matrix (ECM) space leads to cognitive impairments, supporting this mechanism. The introduction of membrane fusion blockers into the postsynaptic cell cytoplasm that attenuates long-term potentiation (LTP), a correlate of behavioral motor activities in response to memory retrieval, provides further support. The lateral spread of activity through the inter-postsynaptic membrane is capable of contributing to oscillating neuronal activity at certain neuronal orders. At the resting state these oscillations provide sub-threshold activation to many neurons at higher orders, including motor neurons maintaining them at a low initiation threshold for motor activity.

## Introduction

Neuronal wiring patterns have been examined using simple behavioral paradigms (Asakawa et al., 2008; Bronson et al., 2008; Cardona et al., 2010; Yu et al., 2010), microscopic examinations (Briggman and Denk, 2006; Hell, 2007), and genetic dissections (Luo et al., 2008; Bernard et al., 2009; Arenkiel, 2011) of neuronal circuits (Kohl and Jefferis, 2011). In addition, viral tracing methods, heterologous receptor expression systems, and optogenetic technologies have been used to examine changes in the neural circuitry of adult-born new neurons (Arenkiel, 2011). Even after using these methods, it was not possible to formulate the functional attributes of neuronal circuitry. Blood-oxygenation level-dependent (BOLD) signal sequences in functional magnetic resonance imaging (fMRI) studies (Logothetis, 2008; Rossier, 2009; Dosenbach et al., 2010) require a supplementary mechanism for the delay-corrected voxel-signals to explain the formation of higher brain functions. Even though the locations of corresponding neurons and their local network were studied by using *in vivo* two-photon calcium imaging followed by electron microscopical examination (Bock et al., 2011), the results are insufficient to explain their functional roles. This has left a huge gap in our understanding about the relationship between neuronal activity and higher brain functions. In addition, different network connectivity analyses have found that similar networks become activated during different tasks (Dosenbach et al., 2007, 2008; Seeley et al., 2007; Stevens et al., 2007; Demirci et al., 2009), requiring an explanation for the overlap. What additional wiring rules should be operating in unison with the known anatomical wiring that enable the formation of internal sensations of higher brain functions along with behavioral motor outputs?

A large body of experimental evidence demonstrates the firing of specific sets of neurons by one of the stimuli that took part in associative learning. Both experimental (Gelbard-Sagiv et al., 2008; Tye et al., 2008) and computational (Kepecs et al., 2008; Lavigne and Darmon, 2008) studies have shown activity from new sets of neurons during memory retrieval, leading to the understanding that this specific set of neurons represents memories. The current difficulties in explaining how neuronal firing creates higher brain functions have suggested the need to explore mechanisms that can explain cognitive functions (Abbott, 2008; Yuste, 2008) and to discover suitable wiring principles (Abbott, 2008; Yuste, 2008) that may explain what constitutes the internal representations in the brain (Sullivan, 2010). Decoding the internal sensations of higher brain functions requires examining the circuit properties capable of encoding new information and later producing internal sensations along with motor neuron activations. Even though motor functions have been used in assessing memory retrieval in experiments, it is clear that the nervous system creates internal sensations even when all the motor actions are restricted. This makes the formation of internal sensations an obligatory property of the nervous system.

An alternative to the conventional connectome studies (Jarrell et al., 2012) is to examine possible basic building units of the nervous system similar to DNA sequences (Zador et al., 2012). These units are expected to have a supplementary mechanism operating along with the known anatomical circuitry, creating internal sensations concurrent with motor neuron activation that execute motor activities. On a functional level, these operations should facilitate beneficial interactions of the system with the environment. We expect the simultaneous arrival of multiple sensory inputs from a nearby item to create specific re-activatible changes in the nervous system. This is expected to facilitate the creation of the semblance of the remaining sensations from the item at the moment when the fastest travelling sensory stimulus reaches the animal when the animal moves away from the item. In the same way, if the animal is close to the item, the arrival of one of the associatively learned stimuli should evoke semblances of the remaining properties of the item. Re-activatible changes taking place at the time of associative learning are likely to occur at locations where different sensory pathways converge after a certain number of orders of neurons; for example, the hippocampus. These re-activatible changes should be able to concurrently activate motor neurons and create effective behavioral motor responses.

## Circuit properties for evoking internal sensations

**Figure 1:**
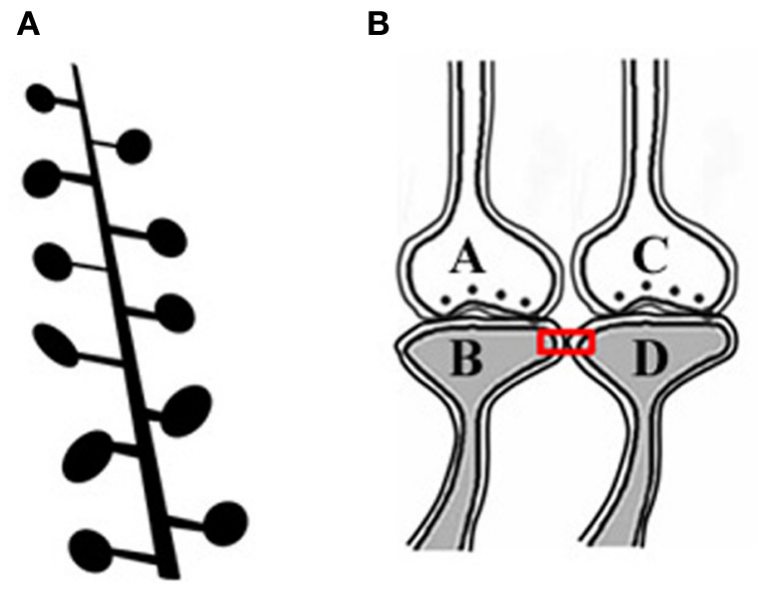
**Adjacent dendritic spines and inter-spine space. (A)** Cartoon showing dendritic spines (postsynaptic membranes) on a dendritic branch of a neuron typically seen by Golgi staining. Presynaptic terminals are not made visible by Golgi staining. Note that the inter-spine distances are larger than the spine head diameter (Konur et al., 2003). The space between the dendritic spines is occupied by extracellular matrix space, glial cells, axonal (presynaptic), and dendritic (postsynaptic) terminals. **(B)** Closely located postsynaptic membranes that are simultaneously activated both during associative learning and LTP induction. A functional LINK (shown by the red rectangular box) is expected to form between the postsynapses as a function of the simultaneous arrival of activity at these postsynapses. A and C are presynapses and B and D are their corresponding postsynapses.

The artificial stimulation of an intermediate order of neurons produces various sensory hallucinations (Selimbeyoglu and Parvizi, 2010), the complexities of which gradually increase as the location of these stimulations moves toward the higher neuronal orders. This lateral entry-induced hallucination of receiving sensory input can be considered an intrinsic property of the system. From this property, we can infer that the naturally present operational mechanism that gets activated during associative learning can be re-activated by the cue stimulus for evoking the internal sensation of the sensory properties of the previous associatively learned item. It is reasonable to assume that the perception in hallucinations and the internal sensation of retrieved memories belong to a spectrum of internal sensations depending on the strength of their formation. Since such changes are expected to occur at the locations of convergence of sensory inputs, (for example, the hippocampus), we examined changes occurring at such locations. The lateral entry of activity from the cue stimulus is hypothesized to induce the internal sensations of the remaining sensory qualities of the item. The most suitable cellular location for normal lateral entry gates should be a location where activity does not flow in a retrograde direction after crossing the synapses. This makes the postsynapses (postsynaptic membranes or dendritic spines or spines) (Figures 1A,B) ideal locations.

**Figure 2:**
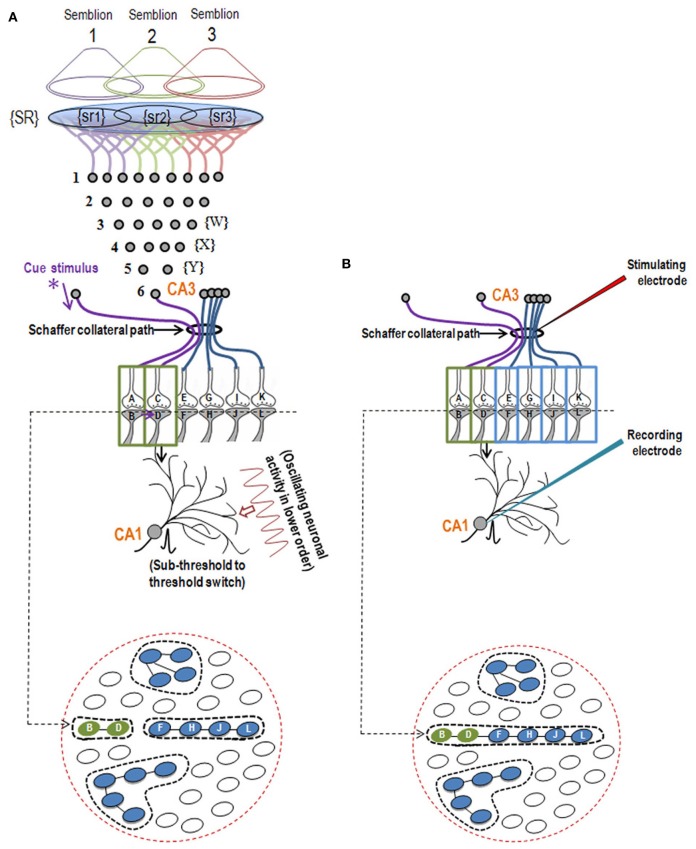
**Illustration showing the structural mechanism of formation of internal sensation of memory and its relationship with a possible mechanism of LTP. (A)** During memory retrieval, a cue-stimulus reaching presynapse A depolarizes its postsynapse B, re-activates the hemi-fused inter-postsynaptic membrane and activates postsynapse D, evoking a cellular illusion of an action potential reaching latter's presynapse C. In normal conditions, an action potential reaches presynapse C when the CA3 neuron is activated. Sensory identity of the semblance of activity occurring at the postsynapse D consists of inputs from the set of neurons {Y} that synapse to the CA3 neuron. The set of neurons {Y} are normally activated by inputs from a set of lower order neurons {X}. The set of neurons {X} in turn are activated by a further large set of its lower order neurons {W}. Continuing this extrapolation toward the sensory level identifies a set of sensory receptors {SR}. {sr1}, {sr2}, and {sr3} are subsets of {SR} and are capable of independently activating the CA3 neuron. Hypothetical packets of sensory stimuli activating sensory receptor sets {sr1}, {sr2}, and {sr3} are called semblions 1, 2, and 3, respectively. The activation of the postsynapse D by the cue stimulus can lead to the virtual internal sensation of semblions 1, 2, 3 or an integral of them. A CA1 neuron (place cell in the context of spatial memory) is shown to receive sub-threshold excitatory postsynaptic potential (EPSP) from oscillating neuronal activities of its lower order neurons. Cue stimulus-induced activation of postsynapse D reaches the soma of its neuron in the CA1 region. If the CA1 neuron receives a baseline summated EPSP short of one EPSP to trigger an action potential, then the additional EPSP arriving from the postsynapse D can add to sub-threshold EPSP, inducing an action potential in the CA1 neuron, resulting in its concurrent activation during memory retrieval; this CA1 neuron will not otherwise be activated in the absence of prior associative learning. This can explain place cell (CA1neuron) firing occurring concurrently with spatial memory retrieval. Bottom Panel: Cross-section through the postsynapses showing a newly formed functionally LINKed postsynapses B and D during associative learning. Three other islets are also shown. **(B)** Stimulation of the Schaffer collateral induces LTP by inducing postsynaptic membrane hemi-fusion between postsynapses that belong to islets of postsynapses B-D and F-H-J-L forming a mega-islet B-D-F-H-J-L. A regular stimulus at the stimulating electrode has now an increased probability of reaching the recording electrode through the large number of hemi-fused postsynaptic membranes within the large mega-islet, showing a potentiated effect when recorded from the CA1 neuron. Neuronal orders from 1 to 6 are numbered from the sensory receptors. Bottom Panel: Cross-section of an area containing the newly formed mega-islet of functionally LINKed postsynapses B-D-F-H-J-L formed during LTP induction. Two other islets are also shown. {SR}, Set of sensory receptors; {sr}, subset of sensory receptors. If LTP-induced mega-islets include postsynapses B and D, it reduces the specificity of retrieved memories in retrieving memories since spread of activity through different non-specific postsynapses of the islet induces non-specific semblances [Modified from Vadakkan (2011b)].

Since simultaneously-activated adjacent postsynaptic membranes are often apposed to each other at locations of convergence of sensory inputs (for example, the hippocampus), with negligible extracellular matrix (ECM) between them (Harris and Stevens, 1989), we examined the interaction between the postsynaptic membranes. It has been observed that the average inter-spine (inter-dendritic spine) distance is greater than the average spine head circumference (Konur et al., 2003), and adjacent neurons share only a small percentage of their inputs (Ecker et al., 2010). This increases the probability of the dendritic spines of different neurons being apposed to each other. This, in turn, increases the feasibility of certain interactions between specific dendritic spines (postsynapses) (between postsynapses B and D in Figure 1B) during associative learning and is referred to as a functional LINK (capital letters are used to highlight its importance) formation (Vadakkan, 2011b). Additional associative learning will result in more postsynapses becoming functionally LINKed. In a cross-sectional view through the inter-LINKed postsynaptic membranes, they can be viewed as islets of functional LINKs (between postsynapses B-D-F-H-J-L in Figure 2, bottom panel).

After associative learning, when the cue stimulus passes through different neuronal orders, it re-activates the inter-postsynaptic functional LINKs (Figure 1) and instantaneously induces the semblance of sensory inputs arriving at the latter. The basic units of semblances are called semblions (Figure 2A) (Vadakkan, 2011b). The natural integration of semblions occurring at physiological time-scales results in the internal sensation of memories. Depending on the specificity of the cue stimulus, a specific set of inter-postsynaptic functional LINKs gets re-activated and induces specific semblances, enabling the retrieval of specific memories. If integration of the semblances from different locations produces an excessive net semblance, it will allow memories to form even if some of the locations of their formation are damaged. This offers an explanation for the circuit property of transfer of memories from the hippocampus to the cortex, namely, consolidation (Vadakkan, 2011a). Since the cue stimulus re-activates the functional LINKs at sparsely distributed individual synapses at various brain locations, the combined effect of the net internal sensations induced during memory retrieval is expected to produce only a virtual internal sensation. In comparison, the internal sensations of hallucinations occurring during artificial stimulation of intermediate orders of neurons (Selimbeyoglu and Parvizi, 2010) should induce denser net semblances, producing a compelling sense of reality. Similarly, perception can be viewed as semblances formed based on previous associative learning. From Figure 2A, it can be seen that neither the physical presence of the neuron marked CA3, its lower orders of neurons {Y}, {X}, {W} nor the corresponding sensory receptors are required to evoke the cellular hallucination (semblance) at postsynapse D. This can explain how the internal sensation of phantom limb is formed.

Reversible as well as stabilizable properties of the inter-postsynaptic functional LINKs make it feasible to view different types of memories as a continuum of the same process occurring at different time-scales, depending on the number of re-activatible units present at the time of memory retrieval. The involvement of previously-formed re-activatible basic operational units explains the ease of related learning. In the case of repetition of a specific associative learning event, related learning or learning between items that activate the same sensory receptor subsets, the newly formed inter-postsynaptic functional LINKs will be maintained long-term through stabilization by certain factors. Retrieval of memories will also maintain inter-postsynaptic functional LINKs. This will enable the maintenance of memories for a long period of time. If the functional LINKs are not re-activated or the stabilizing factors are lost, it will lead to the reversal of inter-postsynaptic functional LINKs, causing memory loss. When the re-activated set of inter-postsynaptic functional LINKs are distributed sparsely at higher neuronal orders, then the net semblances induced from these locations can provide an internal sensation for the specific key features of the item whose memories are retrieved.

## Motor activities concurrent with semblance formation

The lateral spread of activity through inter-postsynaptic functional LINKs can contribute to the horizontal component responsible for the neuronal oscillations at certain neuronal orders (Vadakkan, 2012a). Continuous baseline activity of these neurons causes certain neurons at higher orders to receive sub-threshold summated excitatory postsynaptic potentials (EPSPs), short of eliciting an action potential. As the cue stimulus activity moves toward these higher neuronal orders, additional EPSPs through the re-activated functional LINKs are added to the net EPSPs, allowing it to cross the threshold for eliciting an action potential. Experiments that continuously recorded extracellularly from the CA1 neuronal layer in moving animals have shown that certain CA1 neurons specifically fire (elicit action potential) when the animal reaches specific locations within the field. These cells are called place cells (O'Keefe and Dostrovsky, 1971). Continuous oscillatory neuronal activity at lower neuronal orders provides sub-threshold activation (just a few EPSPs short of an action potential) to some of the CA1 neurons. Therefore, the addition of a few EPSPs arriving from specific cue stimuli from the environment (spatial cue) will be sufficient to fire an action potential in these CA1 neurons when animals reach specific locations within the field.

The same mechanism explains the activation of specific neurons in different regions of the brain during memory retrieval (Gelbard-Sagiv et al., 2008); the current thought is that this activation encodes specific memories. Some of these neurons that are fired concurrent with the arrival of the cue stimulus are motor neurons responsible for motor outputs. Even though all the memory studies have been carried out by measuring the behavioral motor outputs, it is reasonable to assume that the internal sensation of memories of specific items are correlated to the behavioral motor activity resulting from the activation of those motor neurons.

The firing neurons that contribute to the oscillating neuronal activities re-activate a large non-specific set of previously-formed normal functional LINKs (that represent the sensory properties of the items and events from the environment) at higher neuronal orders. The integral of the resulting non-specific set of semblances was hypothesized to provide a framework for consciousness (Vadakkan, 2010a), a baseline requirement for nervous system functions. Blocking synaptic transmission or inducing changes in the oscillatory waveforms during sleep (Massimini et al., 2005) or anesthesia (Llinas and Steriade, 2006) prevents the formation of internal sensations and concurrent motor activities.

## Reversible wiring for inter-postsynaptic functional LINKs

Inter-postsynaptic functional LINKs can operate as a universal mechanism, provided they can be quickly reversed back to independent membranes (explaining working memory as the net semblances formed from the re-activation of the transient inter-postsynaptic functional LINKs before they reverse) or stabilized as hemi-fused inter-postsynaptic membranes for a long period of time (explaining long-term memories). What ideal properties can allow the inter-postsynaptic functional LINKs to operate between two postsynaptic membranes? Even though the spread of neurotransmitters to neighboring synapses (Coggan et al., 2005; Fernandes et al., 2010), 2-amino-3-(5-methyl-3-oxo-1,2-oxazol-4-yl) propanoic acid (AMPA) receptor trafficking (Makino and Malinow, 2009) and ephaptic coupling (Anastassiou et al., 2011) can be regarded as candidate mechanisms occurring between the postsynapses, they lack either the specificity or the time-scales of formation and re-activation or the time-dependent reversibility that are required features for the inter-postsynaptic functional LINK.

At this juncture, we examined disease states that produce symptoms of loss of function, from which the structure-function aspect of the operational mechanism of inter-postsynaptic functional LINKs can be derived. From factors that can disturb memories, it should be possible to understand the nature of the normal operation of the functional LINKs. Once identified, the mechanism can be theoretically tested for its suitability to explain most of the previous experimental findings in all the related fields. Furthermore, if non-specific inter-postsynaptic functional LINKs occur at certain neuronal orders, they are expected to cause cognitive deficits via the formation of non-specific semblions, the activation of a new set of neurons, hallucinations resulting from semblances connecting features of different previously associatively learned items and changes in oscillatory neuronal activities resulting in changes in consciousness. Since all these features are seen in schizophrenia, we examined this disease in detail (Vadakkan, 2012b). Since a large number of previous studies show lipid membrane composition changes in schizophrenia, many of which were explained by chromosomal deletions involving proteins in lipid metabolic pathways, possible changes at the postsynaptic lipid membranes were examined. Significant prevention of the progression of the prodromal stage to schizophrenia through dietary essential fatty acid (EFA) supplementation in a double-blinded randomized control trial (Amminger et al., 2010) and other similar studies indicate that pathological inter-postsynaptic functional LINKs become reversible at the prodromal stage. The non-reversibility of the fully manifested disease state with EFA indicates that the reversible mechanism becomes non-reversible over time. We found that time-dependent irreversibility of the phenomenon is possible when there is a physical interaction between the postsynaptic membranes.

**Figure 3:**
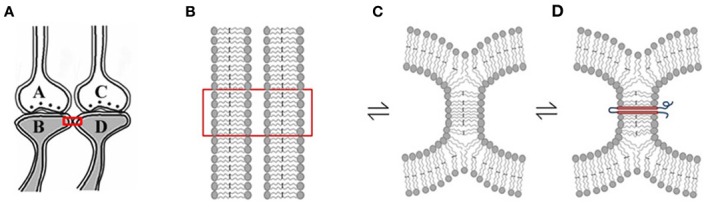
**Rapidly reversible, yet stabilizable hemi-fusion between the postsynaptic membranes of different neurons. (A)** Closely located postsynaptic membranes that are simultaneously activated both during associative learning and LTP induction. A and C are presynapses and B and D are their corresponding postsynapses. **(B)** The boxed region in Panel **(A)** is expanded to show adjacently located postsynaptic cell membranes. **(C)** Simultaneous activation of the postsynapses both during learning and LTP induction can result in instantaneous hemi-fusion between the postsynaptic membranes. If not re-activated, most of these hemi-fused membranes reverse back to independent membranes, making them transient. Note the reversible symbol between different stages of membrane hemi-fusion. **(D**) Repeated formation of the same hemi-fused inter-postsynaptic membrane segment will lead to its stabilization as a homeostatic cellular process. A trans-membrane protein makes the hemi-fused area temporarily stable, depending on the life-span of the protein or its ability to shift away from the hemi-fused area by lateral displacement [Modified from Vadakkan (2012b)].

**Figure 4:**
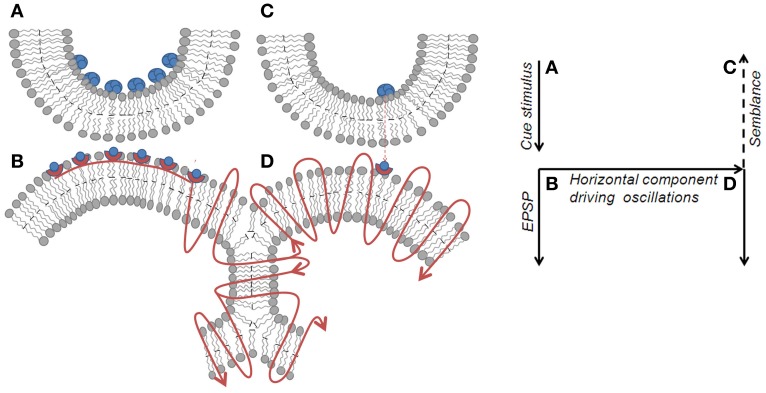
**Spread of activity through an inter-postsynaptic functional LINK. Left panel:** Diagram showing the spread of action potential induced EPSP from postsynapse B through the hemi-fused inter-postsynaptic membrane segment toward the opposite postsynapse D (shown by the curved lines with arrows pointing in the direction of spread of action potential). Unidirectional chemical transmission at the chemical synapses and unidirectional quantal release of single synaptic vesicles (in blue-filled circles) from presynapses inducing miniature EPSP (mEPSP) at the postsynapses sets the stage for the systems feature of semblance formation. When activity arrives at postsynapse D laterally through the hemi-fused area from postsynapse B, it induces a cellular hallucination (semblance) at postsynapse D that the activity is coming from its presynapse C. This is viewed as a systems property. In addition, the lateral entry of activity through the hemi-fused inter-postsynaptic membrane segment provides the horizontal component responsible for oscillating neuronal activity, a required systems property for semblance formation. **Right panel:** Diagram showing the major effects of the re-activation of inter-postsynaptic functional LINKs. The lateral direction of the propagation of activity contributes to the horizontal component responsible for oscillatory neuronal activity. The formation of the semblance is a system property and depends on the frequency of oscillations. Right panel represents the direction of flow of functions in the left panel. A–B is the synapse at which cue stimulus arrives. B–D is the location of inter-postsynaptic functional link. D is the postsynapse at which semblance is formed as a system property.

Rapidly reversible membrane hemi-fusion has been observed extensively in biological systems (Melikyan and Chernomordik, 1997; Kozlov et al., 2010). Since membrane hemi-fusion is dependent on lipid composition, particularly the exchangeable sn2 and sn3 positions of the fatty acid structure, it is reasonable to argue that the derivatives of EFA become incorporated into the membranes, prevent them from forming non-specific inter-postsynaptic membrane hemi-fusions and stop the conversion of the prodrome state (Cannon et al., 2008) to schizophrenia (Amminger et al., 2010). Membrane hemi-fusion that can be temporarily and permanently stabilized through the insertion of trans-membrane proteins (Figure 3) can function as re-activatible gates, meeting the requirements of the functional LINKs. The progression of the prodromal state to the disease state where it becomes non-reversible with EFA supplementation can be explained by the insertion of trans-membrane proteins across the hemi-fused inter-postsynaptic membrane segments (Figure 3) (Vadakkan, 2012b). EPSP can spread through the hemi-fused inter-postsynaptic membrane segment to the functionally LINKed postsynaptic membrane (Figure 4) both to induce semblance formation as a system property and to simultaneously allow this EPSP to spread to its neuronal soma (Note: hereafter, inter-postsynaptic functional LINKs and hemi-fused postsynaptic membranes are used interchangeably).

## Obstructions to hemi-fusion lead to memory defects

Some of the herpes simplex virus-1 (HSV-1) glycoproteins can induce the formation of membrane hemi-fusion by getting inserted into the host membranes (Subramanian and Geraghty, 2007). Since these pathological membrane hemi-fusions are expected to form very non-specifically, neurons infected with HSV-1 can lead to severe cognitive defects as seen in herpes simplex encephalitis. This provides a feasible mechanism for the role of reversible membrane hemi-fusion in the operations for cognitive functions. Similarly, the accumulation of insoluble biochemical intermediates in the ECM space between the postsynaptic membranes (for example, deposition of amyloid proteins in Alzheimer's disease) can explain a mechanism that prevents membrane hemi-fusion at specific inter-postsynaptic membrane locations. This explains the patho-physiology of the cognitive defects in these disorders.

## LTP and semblance formation

**Figure 5:**
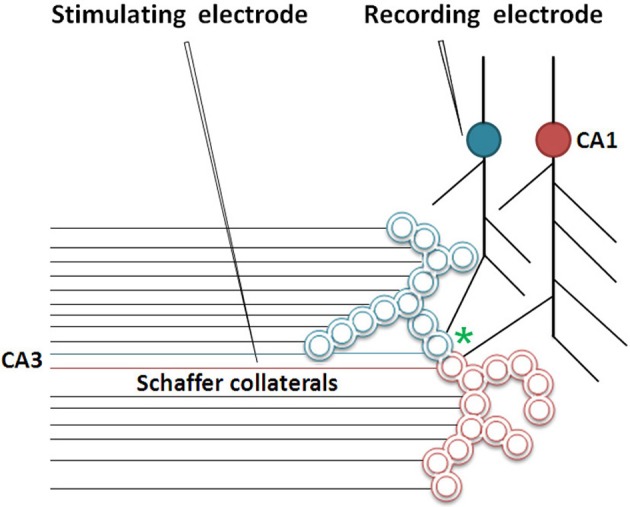
**Cartoon showing the role of inter-postsynaptic membrane hemi-fusion in LTP.** The stimulation of a large number of Shaffer collateral fibers during LTP induction results in hemi-fusion between two postsynapses that belong to two large islets of functionally LINKed postsynapses (in green and red; Note that the double-walled circles are cross sections through the postsynapses as demonstrated in Figure 2). The critical location of hemi-fusion is shown by an asterisk. This results in the merger of these islets to form mega-islets. Following the LTP induction, a regular stimulus at the stimulating electrode induces EPSPs at the postsynapses belonging to both the islets. The recording electrode that records potentials arriving through the blue-colored islet of LINKed postsynapses will start recording the cumulative EPSPs arriving from both the red and blue colored postsynapses of the newly formed mega-islet [Modified from Vadakkan (2010b)].

Studies of the patient H. M. (Scoville and Milner, 1957) revealed that the patient was unable to make any motor expression indicative of experiencing the internal sensations of retrieved memories of associatively learned items or events during a certain period of time prior to the surgical removal of H.M's hippocampi. This case study led to electro-physiological experimentations using isolated rodent hippocampi. The application of an initial brief repetitive stimulation at the axonal regions of the CA3 layer of neurons (Schaffer collaterals) in the hippocampal slices induced a potentiated effect at the CA3-CA1 synapses in response to a regular stimulus applied at the same location at a later time. This was observed by recordings from the CA1 region and is called long-term potentiation (LTP) (Bliss and Lomo, 1973). Following this finding, a large number of studies have shown correlations between behavioral motor outputs indicative of memory retrieval and LTP (Morris et al., 1986, 2003; Whitlock et al., 2006). Such a correlation is possible if similar changes can take place at a location between the site of stimulation (a group of Shaffer collaterals) and collection of responses (one CA1 neuron) during both associative learning and LTP induction (Figure 2B). LTP induction activates bundles of axonal fibers of the CA3 neurons (Schaffer collaterals) and can cause hemi-fusion between large numbers of postsynaptic membranes (dendritic spines) of the CA1 neurons. A normal stimulus at the same stimulating location can then travel through these hemi-fused postsynapses and arrive through a large number of dendrites of a given CA1 neuron, resulting in the recording of a potentiated effect from the latter's soma (Figure 5). The formation of inter-postsynaptic functional LINKs by membrane hemi-fusion both during associative learning and LTP induction provides a feasible explanation for the observed correlation. The reversal of the membrane hemi-fusion can explain the waning of recorded LTP over time and provides a comparable cellular explanation for the loss of memory over time.

Inputs from different sensations reach thalamic projection neurons whose axonal terminals (presynapses) synapse with postsynapses (dendritic spines) of the neurons in the lateral amygdala. We anticipate functional LINK formation between the postsynapses of different lateral amygdala neurons. In patch-clamp experiments following fear conditioning, an example of associative learning, when thalamic afferents are stimulated to measure the EPSPs at the pyramidal neurons in the lateral amygdala, an increase in the amplitude of the AMPA current is observed (Tye et al., 2008). This can be explained as the result of the arrival of additional AMPA currents through the functionally LINKed postsynapses induced during learning (Figure 5). Following fear conditioning, recordings from slices of the amygdala show an increase in miniature EPSP (mEPSP) amplitude (Tye et al., 2008). It is generally interpreted that an increase in mEPSP amplitude corresponds to an increase in the number or function of AMPA receptors (Malenka and Nicoll, 1999), one of the glutamate receptor subtypes. Based on the present work, the increase in mEPSP amplitude can be explained as a function of the additionally measured AMPA channel currents from the functionally LINKed postsynapses (formed during fear conditioning) reaching the patch-clamped neuron (Tye et al., 2008). The formation of functional LINKs between the postsynapses (dendritic spines) of the recording and other neurons may provide the route for the spread of mEPSPs.

N-methyl D-aspartate (NMDA) receptors of the excitatory neurotransmitter glutamate have been shown to be necessary for behavioral motor activities indicative of memory retrieval (Morris et al., 1986), the induction of LTP (Collingridge et al., 1983), and the activation of specific neurons that fire when the animal reaches a specific place in the field (place cell firing) (Kentros et al., 1998). This can be explained by the requirement for cue-induced synaptic activation (synapse A–B in Figure 2) that will then re-activate the functional LINK that induces both the formation of the internal sensation of memory and provides the additional EPSPs required for the activation of sub-threshold-activated neurons. Severe defects in memory and consciousness occur when the NMDA receptors are blocked by auto-antibodies in NMDA receptor antibody encephalitis (Dalmau et al., 2008), demonstrating that cue-induced activation of postsynapses followed by the re-activation of the inter-postsynaptic functional LINKs are essential steps. Further support comes from the previous report that synaptosomal-associated protein (SNAP) inhibitors block membrane fusion and attenuate LTP (Lledo et al., 1998). The effective target of this inhibition is likely taking place at the level of inter-postsynaptic membrane hemi-fusion. Additional evidence is the observation of the possible structural changes from hemi-fusion between the adjacent postsynaptic membranes in the electron microscopic pictures [Figures 2B and 4D in Burette et al. (2012) and Figure 2 in Harris and Stevens (1989), He et al. (1998), Sirvanci et al. (2005)], even though the resolution of the images is limited.

## Necessary conditions for semblance formation

The formation of semblances is viewed as a property of a system in which the lateral entry of activity through the inter-postsynaptic functional LINKs enables its formation at the opposite postsynapse while simultaneously providing the horizontal component responsible for the oscillating neuronal activity. The necessary condition for evoking the semblance of activity from the presynaptic terminal C when postsynapse D (in Figure 2A) is activated by the lateral entry of activity through the inter-postsynaptic functional LINK is that postsynapse D should otherwise be normally activated by its presynapse C (in Figure 2A) in a continuous manner. Continuous quantal release from the presynaptic synaptic vesicles even during periods of rest provides regular arrival of miniature potentials at the postsynapses, which is recorded as mEPSPs or “minis” (Figure 4). The fact that it is very difficult to block mEPSPs “even in experimental conditions” indicates that it is a highly conserved default operation of the system. Another necessary condition is the maintenance of oscillatory neuronal activity. The finding that electrical stimulation of the visual cortex produces a visual percept (phosphene) only when high-frequency gamma oscillations are induced in the temporo-parietal junction (Beauchamp et al., 2012) emphasizes the role of oscillating neuronal activity as a system requirement for semblance formation for creating internal sensations.

## Possible role of dendritic excrescences and recurrent collaterals at the CA3 neuronal order

**Figure 6:**
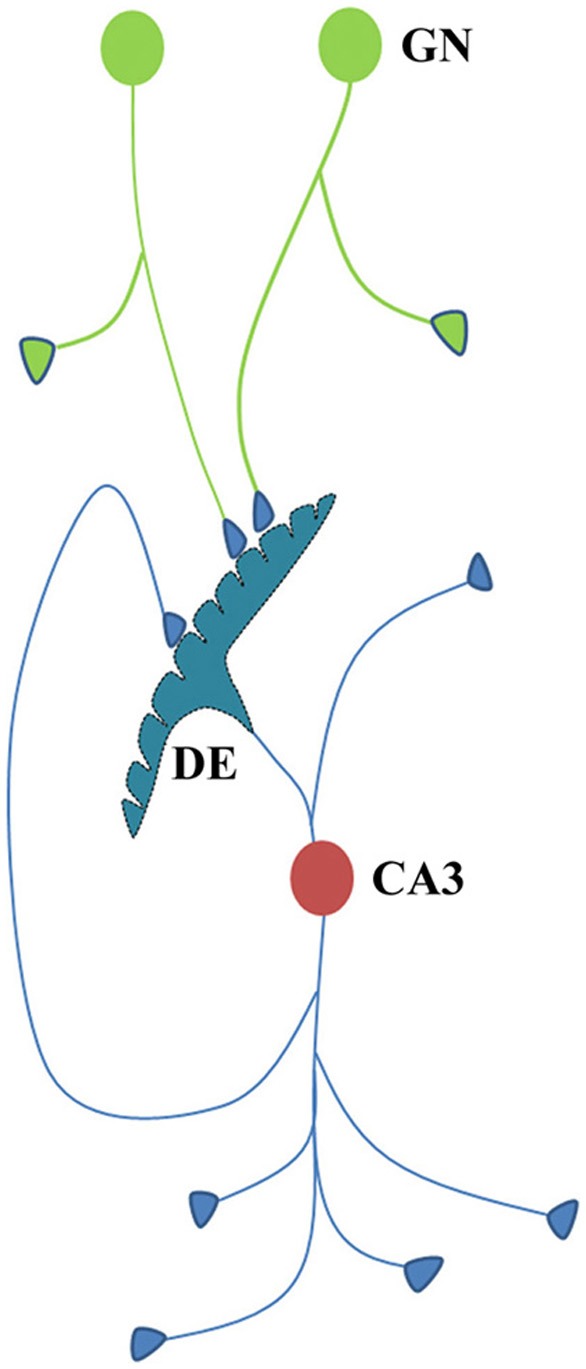
**Cartoon showing different connectional features of CA3 neuron.** Dendritic excrescences present at the dendritic tree of the CA3 neuron (cell body in red) consist of structurally fused postsynaptic membranes. More than thirty fused spine heads have been reported (Bronson et al., 2008). Dendritic excrescences are also reported across different species. In addition, some of the default motor activity in response to the cue stimuli should also be determined by the output neuronal activity from the CA3 neurons. As some of the axonal terminals of the new granule neurons (cell body in green) are likely to synapse with some of the postsynaptic membrane segments of the excrescences, the resulting spread of activity across the excrescences evokes semblions from the neighboring postsynaptic membrane segments that represent the physical properties of the environment. CA3 neurons also have recurrent collaterals that can induce repeated induction of semblances. Formation of semblances (Figure 2) is not drawn in this diagram. GN, granule neuron; DE, dendritic excrescence.

If associative learning between sensory stimuli that pertains to the physical properties of items from the environment constantly arrives at the hippocampus, where sensory inputs converge, it is reasonable to anticipate that evolution must have tried to conserve those functional LINKs most probably in the form of structural LINKs. Dendritic excrescences formed by the fusion of postsynaptic membranes at the closely located dendrites of individual CA3 neurons of the hippocampus (Chicurel and Harris, 1992; Gonzales et al., 2001; Murakawa and Kosaka, 2001) likely to represent evolutionarily maintained inter-postsynaptic functional LINKs resulting from obligatory associative learning between the sensory stimuli from fixed physical properties of the items in the animal's environment and the relationship between various items based on their physical properties (Figure 6). Therefore, a set of functional LINKs for a given nervous system is likely unique to the animal's physical environment. Based on the present work, activity arriving from any of the many presynaptic terminals of an excrescence will induce the semblance of activity at the neighboring postsynaptic membrane segments of the excrescence depending on the spread of activity. Moreover, when the extrapolation of semblances from a higher-order postsynapse reaches the CA3 neuron excrescence (see Figure 2A) it must include all the semblances from all the postsynapses at the excrescence. This includes all the related (previously associatively learned) sensory inputs from the environment, depending on the physical properties of the items in the environment. The induction of semblances at the excrescence also depends on the relationship with oscillations in the neighboring neuronal orders. An additional feature of CA3 neurons is the presence of recurrent collaterals from their axonal terminals that synapse on to themselves, facilitating the re-entry of their own activity (Figure 6). Recurrent collaterals synapsing to the excrescences can provide continuous semblances for the sensory inputs related to the physical properties of the environment, the nature, and effects of which need to be explored.

## Effect of incorporation of new neurons in an intermediate neuronal order

**Figure 7:**
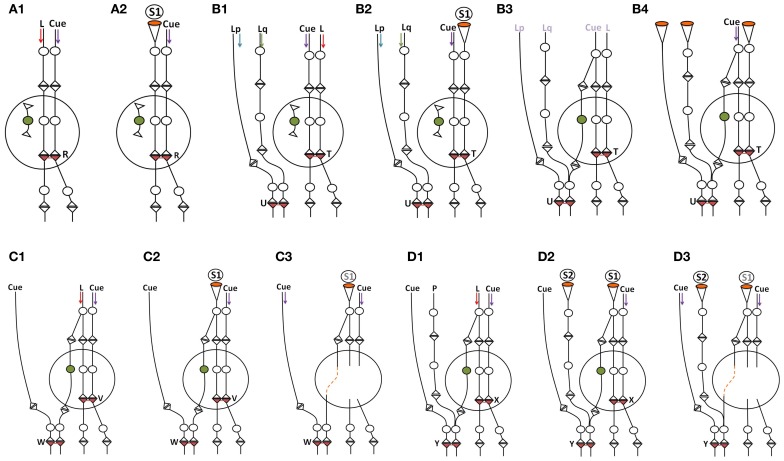
**Schematic diagram showing the effect of incorporation of new neurons within the neuronal circuitry.** The incorporation of thousands of granule neurons on a daily basis can result in the introduction of new connections between the neurons in the entorhinal cortex and the CA3 neurons. Following learning, the establishment of new neuronal connections can evoke unrelated semblances in response to a specific cue stimulus since the new neuronal connections necessitate the inclusion of the semblances evoked from the unrelated pathways to which it gets connected. This can reduce net memories. However, repetition of the associative learning, related learning, or the simultaneous activation of receptor pairs originally activated during the associative learning can lead to the formation of new inter-postsynaptic membrane hemi-fusions at higher neuronal orders, increasing net semblances for memories. The following are examples of some of the conditions that influence net semblance for memory. Note that the diagrammatic expression of the extrapolation of the semblance (see Figure 2) is simplified here. **(A1,A2)** Learning and memory retrieval in a circuitry before the incorporation of new neuron. **(A1)** Learning before the incorporation of new neurons in the hippocampal granule layer. L, Item to be learned. An inter-postsynaptic functional LINK is formed at location **R** when activity from the cue stimulus (one of the associatively learned stimuli) and the item to be learned reach the apposed postsynapses at this location. **(A2)** Memory retrieval before the incorporation of new neurons in the hippocampal granule layer. Here, a semblance is formed from the re-activation of an inter-postsynaptic functional LINK within the hippocampus. **(B1–B4)** Reduction in memory due to dilution of semblances following new neuron connections in the circuitry in the absence of repetition of learning or related learning or simultaneous activation of the sensory receptor pair involved in the associative learning. **(B1)** Associative learning before the addition of a new neuron. Note the presence of an extra-hippocampal inter-postsynaptic functional LINK at location **U** formed from associative learning between items Lp and Lq. **(B2)** Memory retrieval by the cue stimulus induces specific semblances at location **T** immediately following learning (before the incorporation of the new neuron in the circuitry). **(B3)** A new granule neuron is incorporated into the circuitry in the absence of repetition of learning or related learning or simultaneous activation of the receptor pairs. The labels are given in light colors to denote that there are no active stimuli at this time point. **(B4)** Memory retrieval following the incorporation of a new neuron by the cue stimulus induces unrelated semblances through the inter-postsynaptic functional LINK at location **U** that reduces memory. **(C1–C3)** Learning and memory retrieval after the incorporation of a new neuron. **(C1)** Associative learning after the incorporation of new neurons in the hippocampal granule layer. Note the formation of an inter-postsynaptic functional LINK within the hippocampus at location **V**. Also note that while the input pathway from the item to be learned passes through the hippocampal new granule neuron to reach the higher neuronal orders, the inputs from the cue stimulus bypasses the hippocampus to reach the higher neuronal orders and forms an inter-postsynaptic functional LINK at the location **W**. **(C2)** During memory retrieval, after the incorporation of new neurons in the circuitry, the net semblance is stronger than before the introduction of the new neuron. **(C3)** Memory retrieval after the removal of the hippocampus. Since semblance formation does not require the physical presence of a connection toward the postsynapses at which it is formed, semblance from the location **W** is evoked as **S1**. This is similar to the formation of the phantom limb phenomenon. Even though the number of inter-postsynaptic functional LINKs increases at higher neuronal orders, the nature of the semblances that are formed will be less specific when the hippocampus is removed. **(D1–D3)** Loss of memories following the removal of the hippocampus. **(D1)** Associative learning between the cue stimulus and the item to be learned. Neurons at the locations of convergence receive a different number of unrelated sensory inputs; for example input from **P**. **(D2)** Backward extrapolation from the postsynapse at which semblance is induced should include all the synaptic inputs through which activity had arrived via the neuron of its presynaptic terminal (see Figure 2), making the pathway from **P** a possibility for semblance formation. Semblance induced at location **Y** by the cue stimulus after the removal of hippocampus induces non-specific semblance **S2,** reducing memory. **(D3)** As the net non-specific semblances **(S2)** induced after the removal of hippocampus become more than the net specific semblances **(S1)**, the net semblance required for specific memory retrieval is reduced [Modified from Vadakkan (2011a)].

The continuous integration of new neurons in the circuitry at the granule neuron layer of the hippocampus introduces new locations of functional LINK formation at higher neuronal orders during the repetition of associative learning. This leads to the formation of more semblions at the time of memory retrieval. Even though the specific features of semblions formed at the hippocampus and cortex are likely to be different, their cumulative effect is expected to strengthen the net semblance. In humans, the continuation of this process for nearly ten years will allow the formation of sufficient semblances from the cortex (locations of secondary and higher levels of convergence of sensory inputs) such that the nervous system becomes capable of retrieving similar memories even when the hippocampi are removed. This explains the process of consolidation of memories (Vadakkan, 2011a) (Figure 7). However, the incorporation of new neurons without the repetition of learning or the activation of the same set of sensory receptors used in previous associative learning events can lead to a reduction in the net semblance (Figure 7). We have previously seen that at the time of learning a very large number of reversible inter-postsynaptic membrane hemi-fusions are formed that can contribute to the large net semblance for working memory. As they reverse over time, the net semblance for memory reduces. The incorporation of new neurons can further reduce memories through the addition of non-specific semblances if there are no repetitions of learning. On the beneficial side, continued incorporation of new neurons along with repetitions of learning or related learning or the simultaneous activation of sensory receptor pairs result in the widely distributed locations of semblance formation with an excess net semblance during memory retrieval (Figure 6).

## Neuronal oscillations maintain low initiation threshold neurons for motor activities

Regions in the brain where the functional LINKs are densely located (for example, the hippocampus) show slow oscillations (Sirota and Buzsaki, 2005; Beauchamp et al., 2012). These regions are expected to have both horizontal and vertical vector components driving these oscillations. The synaptic transmission can provide the vertical component and the spread of activity through the inter-postsynaptic functional LINKs can provide the horizontal component responsible for the oscillatory pattern of neuronal activations (Figure 4). As a result of these oscillations, a large number of neurons and their connected pathways remain activated during rest and during the operation of visual, sensory, motor, language, and cognitive functions explaining the findings in different imaging studies (Cordes et al., 2000; Beckmann et al., 2005; Fransson, 2005; Dosenbach et al., 2007; Seeley et al., 2007). Since the activation of these neurons during baseline oscillations doesn't spontaneously evoke motor activity, it suggests that they are under strong inhibitory or modulatory control. In this context, it is important to note that experiments to electrically stimulate the visual cortex for inducing visual percepts (pressure phosphene) succeeded only when artificial stimulation had evoked high-frequency gamma oscillations in the temporo-parietal junction (Beauchamp et al., 2012). This implicates that the frequency of oscillations determines the intrinsic property of internal sensation induced by the system.

Oscillatory neuronal activity results in the sub-threshold summation of EPSPs at the axon hillocks of a very large number of neurons at the higher neuronal orders. Maintenance of these sub-threshold activated neurons serves an important physiological role by providing “ready-to-fire” neurons. For example, a sub-threshold-activated neuron just short of one EPSP to elicit an action potential is expected to become activated with the arrival of a single EPSP at one of its dendritic spines (postsynaptic terminals) in the dendritic tree through the re-activation of a functional LINK by activity arriving from the cue stimulus. Similarly, sub-threshold motor neurons maintained by continuous oscillatory neuronal activity can have a significant role in central pattern generator functions at different locations of the nervous system and in the initiation and maintenance of locomotion.

## Wiring diagram supporting internal sensations and concurrent motor activities

**Figure 8:**
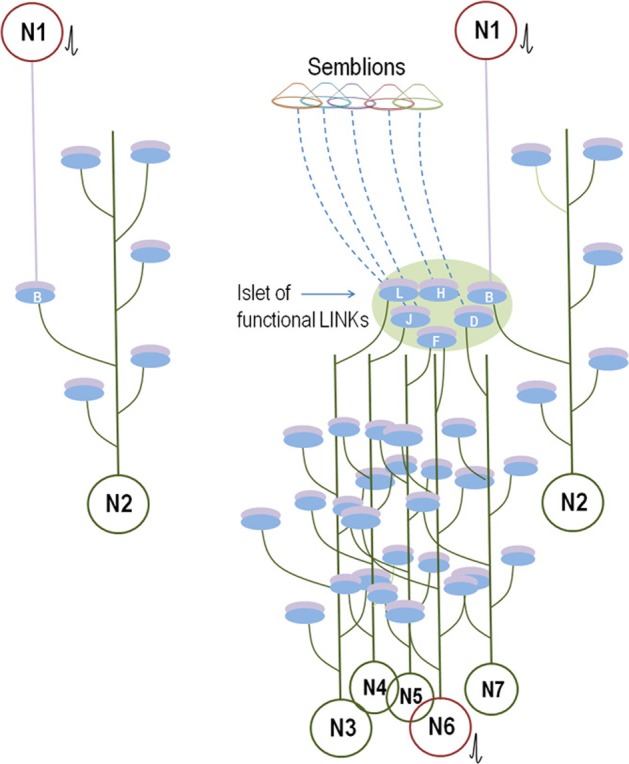
**Comparison between the known anatomical circuitry and the inter-postsynaptic functional LINK-mediated wiring. Left panel:** Synaptically connected conventional neuronal circuit diagram. There is one synaptic connection between neurons N1 and N2. The activation of neuron N1 induces an EPSP at postsynaptic membrane B. Provided neuron N2 is simultaneously receiving EPSPs from other neurons, the sum of which is just one EPSP short for spatial summation to trigger an action potential, then the EPSP arriving at postsynapse B from the activation of neuron N1 will lead to the firing of neuron N2. The contribution of the EPSP from the activation of Neuron N1 toward the temporal summation of EPSPs to elicit an action potential in neuron N2 should also be considered. Otherwise, a single EPSP or a train of few EPSPs reaching at postsynapse B alone may not induce an action potential of neuron N2. **Right panel:** Wiring diagram based on the present work. The activation of neuron N1 activates the inter-postsynaptic functional LINKs between the postsynapses in the islet of functional LINKs (Figure 2, **bottom panel**). The re-activation of postsynapse B that belongs to neuron N2 can provide EPSP and enable neuron N2 to fire an action potential similar to the threshold conditions explained for neuron N2 of the conventional wiring diagram (in the **left panel**). In addition, EPSPs spread to other hemi-fused postsynapses D, F, H, J, and L (depending on the extent of the spread through the islet) that can reach toward their neuronal somata. According to the supplementary rules, a total of six postsynapses are re-activated here, in comparison to only one by the canonical synaptic transmission (**left panel**). This increases the probability for firing of sub-threshold activated neurons in the next order by bringing them toward the threshold for activation. For example, neuron N6 continuously receives (*n* − 1) EPSPs, just short of one EPSP toward either spatial or temporal summation to elicit an action potential. Arrival of the *n*th EPSP from the islet of functionally LINKed postsynapses enables neuron N6 to cross the threshold to elicit an action potential (shown in red). If neuron N6 is a motor neuron, it can evoke motor activity concurrent with the re-activation of the functionally LINKed postsynapses B, D, F, H, J, and L. Activity through these LINKed postsynapses will also evoke semblions for the formation of internal sensations provided these are located at regions of oscillatory neuronal activity. All the neurons in red receive sufficient summated EPSPs and fire action potentials.

The formation of internal sensations depends on the nature of the semblions formed in response to the specificity of the cue stimulus. The lateral spread of activity through the inter-postsynaptic functional LINKs induces physiological oscillatory neuronal activity, which maintains large numbers of sub-threshold activated neurons at the higher orders. The latter are activated by the arrival of one or a few EPSPs and determine the neurons that are activated by the arrival of the cue stimulus (Kentros et al., 1998; Gelbard-Sagiv et al., 2008). Motor activity triggered by these neurons can occur concurrently with the formation of semblances during memory retrieval (Figure 8). By introducing inhibitory control over these neurons, their activation can be utilized for efficient physiological purposes. The nervous system that commands motor actions in response to a cue stimulus immediately receives feedback sensory inputs from the resulting motor activity. These arrive through visual inputs, the activation of vestibular labyrinths, and somato-sensory afferents (superficial sensations and proprioceptors from the joint capsules, tendon, and muscle position sense). The feedback inputs fine-regulate the operations of the system. Thus, the system is getting updated regarding each step of the motor action until the end of its execution.

Since the nervous system has only a finite number of synapses at which inter-postsynaptic functional LINKs can be formed, continuous associative learning using an infinite number of sensory stimuli from the environment results in the sharing of a large number of functional LINKs. Considering that the functional LINKs are a part of the new wiring that occurs during the acquisition of information from the environment, its effects need to be incorporated into the circuitry. Even though it appears to occur only for the duration of time that these functional LINKs exist, a thorough examination can show that their effects on the circuitry at higher neuronal orders may last longer than their own existence.

The stability of the newly formed functional LINK-induced circuitry depends on the repetition of the associative learning that (a) maintains the required molecular changes and may eventually convert them to near-structural LINKs, and (b) incorporates more new neurons in the circuitry to expand the number of functional LINKs from which a large number of combinatorial semblances can be induced for retrieving different memories. Exposure to rare combinations of sensory stimuli will lead to the formation of specific new sets of functional LINKs at higher neuronal orders. The cognitive abilities that depend on the capacity to associatively learn specific patterns of physical properties of various items in the environment are likely to depend on the available unique combinations of postsynapses that can be functionally LINKed. Many functional LINKs are expected to be formed by simultaneous inputs from the environment that depends on the fixed physical properties of the items. It is possible that the functional LINKs get evolutionarily preserved as structural LINKs and are maintained through genetic mechanisms. In a novice nervous system, synaptic neurotransmission, and the spread of activity through innate structural LINKs between postsynapses will be responsible for innate behavioral responses (movement toward the source of food, sucking, and swallowing etc.) required for basic survival needs.

It is anticipated that successful stable memories for an item will have excess of net semblances beyond what is required, so that the system can afford to lose some of the functional LINKs without losing the required minimum net semblances for a specific memory (Vadakkan, 2010b). The brain circuitry is expected to quickly equilibrate with the changes including the effect of functional LINK re-activation, the non-linear integration of semblances, and the cellular changes that maintain stability. Given the constant formation of transient functional LINKs, their reversibility, and the activation of new sets of neurons, the nervous system circuitry is very dynamic at all times. At the instance of the arrival of new combinations of sensory (cue) stimuli, new combinations of internal sensations are created. The extent and complexity of the latter can possibly create complex higher brain functions; for example, emotions.

## Conclusion

The gold standard requirement for the operational mechanism of a complex system is an interrelated framework that can explain almost all its functions. The inter-postsynaptic functional LINKs can provide these requirements, which include the retrieval of memories at physiological time-scales, the consolidation of memories (Vadakkan, 2011a); the ability to support a framework of consciousness (Vadakkan, 2010a), the ease of learning related items; working memory resulting from semblances from all the functional LINKs immediately after learning; deterioration of the strength of memories immediately following learning as the hemi-fused membranes reverse back to their low-energy state independent membranes; the repetition of learning maintaining the hemi-fusions for long periods of time until certain trans-membrane proteins are inserted across them enabling long-term memories; the role of new neurons in losing, improving, and expanding the locations of formation of memories; and the sharing of the mechanism of inter-postsynaptic membrane hemi-fusion in memory with LTP. Since the explanation for the large number of nervous system functions is possible from unitary functional units, the additional rules discussed here should be considered a testable biological mechanism of nervous system functions.

We have presented a supplementary circuit rule-set that can operate in unison with existing circuit rules and provides interconnected frameworks to explain various nervous system functions. It was imperative to make reasonable assumptions to view the formation of semblances as an emergent property of a system having oscillatory neuronal activity at certain neuronal orders. In such systems, the lateral entry of activity re-activating the inter-postsynaptic functional LINKs provides the horizontal component responsible for the neuronal oscillations along with the formation of basic units of internal sensations; namely, semblions. The concurrent formation of semblances and behavioral motor activity that depends on the frequency of neuronal oscillations provides a finely-regulated system. The present work highlights the importance of developing technologies to measure the summated EPSPs from the soma of the neurons, both at rest and during a cognitive operation, as an initial step followed by developing methods to trace the synapses from where they arrive. Verifying the wiring rules by examining the basic structural mechanisms of operations will help us understand additional information regarding the first-person perspective of different higher brain functions.

### Conflict of interest statement

The author declares that the research was conducted in the absence of any commercial or financial relationships that could be construed as a potential conflict of interest.

## Abbreviations

AMPA: 2-amino-3-(5-methyl-3-oxo-1,2-oxazol-4-yl) propanoic acid
BOLD: Blood oxygenation level dependent
CA1: Cornu Ammonis region 1
DE: Dendritic excrescence
ECM: Extracellular matrix
EFA: Essential fatty acid
EPSP: Excitatory postsynaptic potential
fMRI: functional magnetic resonance imaging
GN: Granule neuron
LINK: link (capital letters are used to highlight its importance)
LTP: Long-term potentiation
mEPSP: miniature excitatory postsynaptic potential
NMDA: N-methyl D-Aspartic acid
Postsynapse: Postsynaptic terminal (dendritic spine)
Presynapse: Presynaptic terminal
SNAP: Synaptosomal-associated protein.
